# Peritoneal Immunosurgery: Immunotherapy Augmented Surgery for the Treatment of Peritoneal Cancers

**DOI:** 10.1002/jso.70075

**Published:** 2025-09-17

**Authors:** Ada I. Ozcan, Arianexys Aquino López, Mary K. McKenna, Malcolm K. Brenner, Alastair M. Thompson

**Affiliations:** ^1^ Center for Cell Gene Therapy, Baylor College of Medicine Texas Children's Hospital, Houston Methodist Hospital Houston TX USA; ^2^ Department of Pediatrics Texas Children's Hospital Houston TX USA; ^3^ Department of Molecular and Cellular Biology Baylor College of Medicine Houston TX USA; ^4^ Department of Medicine Baylor College of Medicine Houston TX USA; ^5^ Department of Surgical Oncology Baylor College of Medicine Houston TX USA

**Keywords:** immunotherapy, peri‐operative immunity, peritoneal cancer, peritoneum, surgery

## Abstract

Peritoneal malignancy often indicates disruptions in multiple physiological systems resulting from widespread cancer. The heterogenous origin and dynamic nature of peritoneal cancer make it difficult to treat with standard approaches that fit into guidelines. We describe how successful treatment should address the underlying pathology, the systemic response to surgical treatments and target the immune perturbations that facilitate the establishment and propagation of this multifaceted disease.

Cancer immunotherapy harnesses the immune system to mount therapeutic responses against malignancy with a subsequent goal of establishing a memory response that can prevent recurrences [1, 2]. To establish durable immune responses against cancer, we can use drugs, antibodies, toxins, vaccines, and viruses that engage the immune system against cancer, or employ adoptive cellular therapies to target cancer in ways unavailable to the natural immune system [3]. Current therapeutic approaches include: inducing tumor cell death through monoclonal antibodies against tumor‐associated antigens; supporting innate and adaptive immunity with checkpoint antibodies, vaccines, or cytokines, and utilizing oncolytic virotherapy [4, 5, 6]. Adoptive cellular therapies equip patients with immune cells, natural or engineered, that can replace or even outperform endogenous immune effector mechanisms that would fail to produce a sufficient antitumor response [7]. For greater specificity and efficacy, these immunotherapeutics can be complemented with custom‐designed delivery methods [8].

Peritoneal metastatic disease (PMD) often signifies disseminated disease spanning multiple anatomic compartments, each of which may respond differently to conventional treatments [9, 10, 11]. Effective treatment of PMD requires the recruitment of the immune system to assess and respond to these differing needs. Multifaceted treatment protocols incorporating immunotherapy, when combined with surgery, hold promise for overcoming the challenges we currently face in treating this dynamic disease [2, 12]. in this review we assess the current status of PMD treatment, outline the rationale for integrating immunotherapy with surgery to eradicate PMD, and consider the associated challenges.

## 1 PMD Pathophysiology

PMD has a significantly higher incidence than primary peritoneal malignancy, arising most commonly from ovarian, colorectal, pancreatic, or gastric cancers [9, 13, 14]. The anatomy that allows wide access to critical abdominal organs, the plasticity of the mesothelial cells, and the supportive nature of the submesothelium contrasted with its low capillary density are some of the features that have been proposed to contribute to peritoneal cancer progression in the face of systemic treatments [13, 15].

The parietal and visceral peritoneum lie continuous with the serosal linings of abdominal organs connected to anatomically separate networks of blood vessels [16]. This segregation may allow the portal circulation from the visceral lining to connect with the inferior vena cava via the parietal peritoneum and provide invading cancer cells with a shortcut between the two networks [16]. The peritoneum consists of a superficial monolayer of mesothelium bound by tight junctions and a supportive connective tissue submesothelium [17]. The mesothelium is in direct contact with the abdominal cavity and represents the first line of physical defense against cancer invasion, expressing molecules that inhibit attachment [18, 19]. The tightly‐knit nature of the mesothelium allows higher local concentrations of intraperitoneally administered therapeutics, ensuring their greater efficacy [20]. Mesothelial cells are unique in their ability to undergo epithelial‐to‐mesenchymal transition (EMT) in response to certain stimuli, potentially giving rise to various cell types [21]. EMT can produce cancer stem cells that fuel neoplastic proliferation and contribute to chemotherapy resistance [22].

in contrast, the submesothelium is the immediate continuation of serosal linings from visceral organs [16]. It is rich with collagen fibrils, adipocytes, fibroblasts, blood, and lymph vessels of topographically varying density [17]. This provides ample resources in the microenvironment to sustain cancer cells while also expressing factors that may recruit them [23, 24, 25, 26, 27, 28]. in short, cancer cells that have successfully arrived at the peritoneal surface can re‐establish their microenvironment due to the mesothelium's plasticity and the submesothelium's nutritious nature.

Beyond the additive benefits of addressing resectable tumors with surgery and targeting dissemination with immunotherapy, the integration of these modalities can confer synergistic advantages. Specifically, targeted perioperative manipulations of the immune system can redirect its response towards an antitumor effect that fosters superior surgical outcomes [29]. The immune response to surgery presents valuable opportunities to augment therapeutic efficacy, along with considerable challenges.

## 2 Rationale for Immunotherapies in the Setting of PMD Surgery

The protumorigenic effects of surgery have been simplified into the “seed and soil” hypothesis [30, 31]. Surgical trauma disrupts the tumor microenvironment releasing cells that behave as “seeds” and simultaneously compromises immune function, allowing these cells to evade detection [32]. The compromised Postoperative immune system guided by the proliferative signals essential for tissue repair, can exert a cancer‐supportive influence and make the “soil” more receptive.

The forces that work against a surgeon to favor tumor progression come into effect with the scalpel diving into the dermis, as platelets activated by the incision can attach to circulating tumor cells (CTCs) and shield these “seeds” from immune recognition until they reach their site of metastasis [33, 34, 35, 36]. The proangiogenic growth factors and immunosuppressive cytokines essential to the postoperative wound healing process can inadvertently promote the proliferation of these cancerous foci [37, 38, 39]. As a result, the success of surgical resection can be hindered by the progression of minimal residual disease or malignant transformation of pre‐neoplastic tissue beyond surgical margins [32, 40, 41, 42, 43, 44]. The reported post‐Cytoreductive Surgery/Hyperthermic Intraperitoneal Chemotherapy(CRS/HIPEC) relapse rates of 41% and 69% from Pseudomyxoma Peritonei and colorectal cancer PMD cohorts highlight the need for systemic interventions targeting Postoperative tumor biology [45, 46]. Clinical trials showing reduced Postoperative metastatic progression with endostatin, an innate antiangiogenic factor that is suppressed after surgery, suggest that immunotherapeutic interventions can modulate mechanisms driving postsurgical tumor progression and improve treatment outcomes [37, 47, 48, 49].

The phenomenon of Postoperative tumor progression has been documented in healthy mice after mock surgery [50, 51]. Mock surgery‐treated mice had increased rates of tumor growth and metastatic progression compared to non‐operated mice. The compromised immune system was revealed as the driver of the post‐surgery pro‐tumor response when the adoptive transfer of surgically stressed natural killer (NK) cells into healthy mice resulted in compromised tumor control [52]. Considering the fundamental role the immune response plays in shaping Postoperative treatment outcomes, treatment protocols that do not adequately consider the immune system are prone to fail.

On the other hand, the enhanced local blood flow, paired with increased tumor antigen exposure following surgery, presents a unique opportunity for a competent immune system to recognize and target cancer [2, 53, 54]. If effective immune‐surveillance mechanisms are in place, surgery‐induced antigen exposure can initiate a cascade of reactions that result in long‐term immunologic memory [55, 56]. Unfortunately, the Postoperative immune system is often not in the optimal state to take advantage of this opportunity, and this relatively brief period may play a disproportionately significant role in long‐term treatment outcomes [29, 54]. Strategies that shape and redirect the immune response during this underappreciated period can confer a substantial benefit [29, 55]. These strategies include perioperative treatments with agents ranging from small molecule drugs, cytokines, and oncolytic viruses to the influenza vaccine [57, 58, 59, 60, 61, 62, 63].

Surgically augmented immunotherapies have the potential to minimize on‐target off‐site effects by precisely delivering treatments within the confines of the TME [20, 64, 65]. in addition to systemic treatments, operative delivery of certain therapeutic agents may prove safer and more efficacious by circumventing some of the undesired interactions within the systemic circulation [64]. Peritoneal cancer could be eliminated when we identify the advantages and shortcomings of current immunotherapeutics and design surgically guided protocols to optimize the benefits [66].

## 3 Immunotherapies for Cancers of the Peritoneum

**Figure 1:**
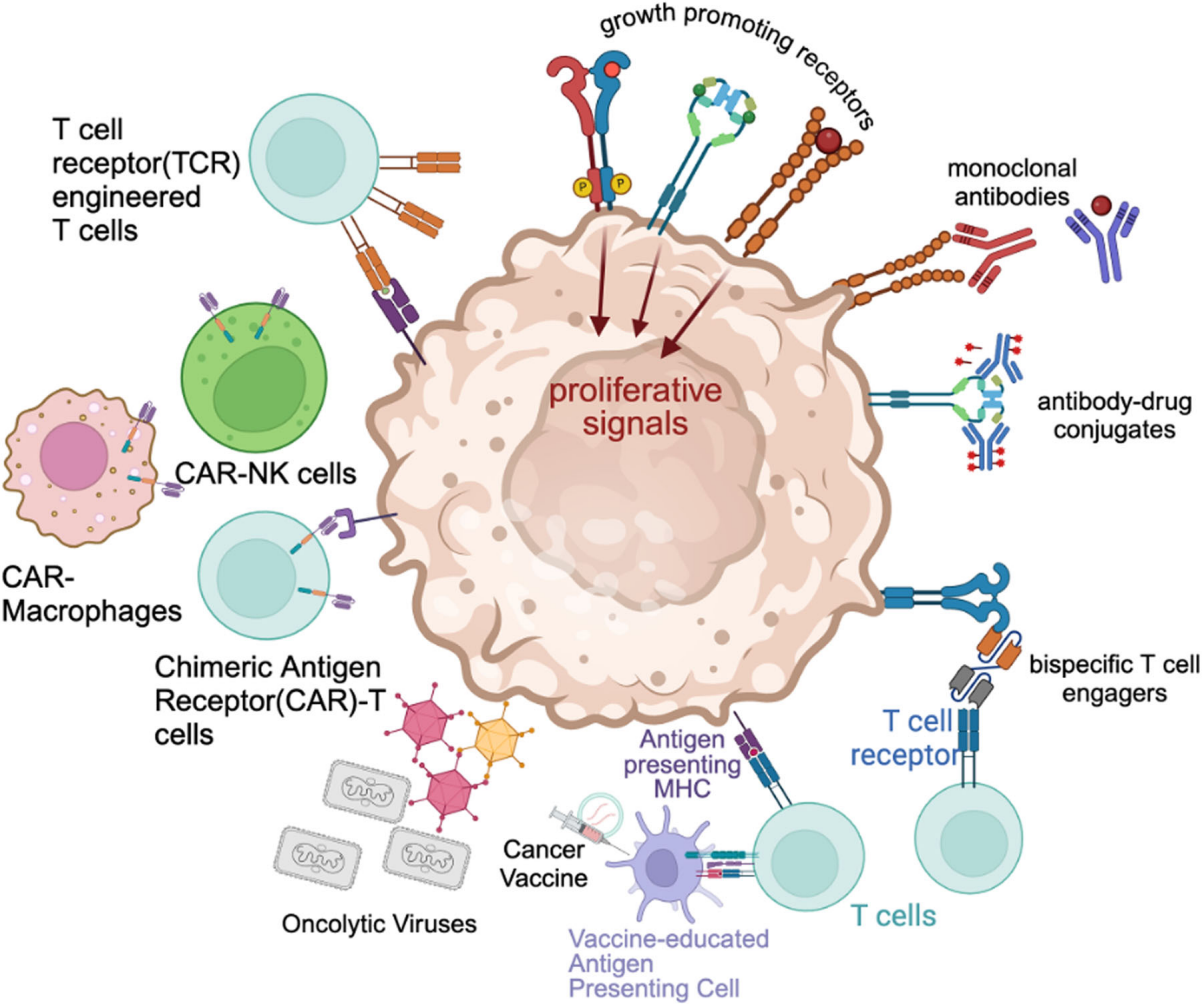
Summary of Reviewed Immunotherapy Modalities.

This review focuses on key aspects of the main immunotherapeutic approaches being considered for the treatment of cancers affecting the peritoneum, with the exception of Immune Checkpoint Blockade (ICB). The revolutionary contributions of ICB therapies to the current landscape of solid tumor immunotherapy are beyond the scope of this article and are extensively reviewed elsewhere [6, 67]. However, the activity of many of the modalities discussed here may well be further augmented by integration of ICBs (Figure 1).

### 3.1 Monoclonal Antibodies

**Monoclonal antibodies (mAbs)** are recombinant, usually humanized, antibodies designed to recognize and bind to molecules associated with malignant disease [68]. For cancer immunotherapy, mAbs are directed toward molecules that promote immunosuppression and facilitate cancer survival [4, 69]. Upon binding to their respective ligands or receptors that support cancer survival, such as growth factor receptors, mAbs block their activation, deprive the cancer cell of growth‐promoting signals, and cause cell death [68]. mAbs also recruit the immune system by complement‐dependent cytotoxicity (CDC) and antibody‐dependent cellular cytotoxicity (ADCC) to induce cell lysis [70, 71]. For more significant cytotoxicity, mAbs can be linked to a cytotoxic agent, referred to as a “payload”, in the form of drugs (antibody‐drug conjugates [ADC]), microbial toxins (immunotoxins) or radionuclides [72, 73]. To enhance immune engagement, a subset of mAbs, including Bispecific T cell Engagers (BiTEs) and Bispecific NK cell Engagers (BiKEs), can simultaneously bind to different epitopes on effector and tumor cells to physically crosslink the two [74, 75, 76].

An overview of the peritoneal structures and mechanisms harboring metastasis can help us better utilize these therapeutics. As cancer cells traverse the abdominal cavity in peritoneal fluid, they must avoid anoikis‐ the subtype of apoptosis triggered by the lack of appropriate cellular attachments‐ and evade lymphatic delivery toward immune recognition [11, 77, 78]. The peritoneum provides protection from both by offering a surface for attachment and hence supports metastatic growth [11, 77, 78]. While mesothelial cells can sometimes deter attachment, local trauma and inflammatory mediators of the post‐surgery response, including reactive oxygen species (ROS), Tumor Necrosis Factor‐α (TNF‐α), and IL‐6, can cause the upregulation of surface adhesion molecules that favor adhesion [79]. Upon attachment, cancer cells can reprogram the mesothelium towards a cancer‐supportive phenotype and utilize the resources provided by the submesothelium to thrive [11, 80, 81]. Therefore, there is a growing emphasis on developing approaches to target adhesion pathways [82]. Despite implicating a multitude of adhesion molecules in the pathogenesis of PMD, only a select few could be modified to alter the disease trajectory. A systematic review of the literature identified the interactions between cancer MUC16, an extracellular mucin, and peritoneal Mesothelin as clinically relevant vulnerabilities of PMD [83, 84, 85]. Mesothelin (MSLN), initially identified as an overexpressed cell surface marker in ovarian cancer, is a glycoprotein normally found on the mesothelial linings of the pericardium, pleura, and peritoneum [86]. MSLN binds to MUC‐16, better known for its epitope CA125, also expressed on ovarian cancer and mesothelioma, promoting cellular adhesion, and facilitating invasive behavior [87]. The N‐linked oligosaccharides of MUC‐16 may mediate this interaction and consequently abrogate the recognition of these glycosylation patterns, possibly with lectins [88, 89]. MUC16 has also been shown to promote disease progression and treatment resistance and protect from NK cell cytotoxicity [90, 91, 92, 93]. MUC‐16 can also serve as a prognostic biomarker guiding patient selection for the treatment of PMD [94].

Compelling results from MSLN knock‐out studies, demonstrating compromised tumor cell growth and invasion upon MSLN silencing, have spurred interest in MSLN as a target for immunotherapy [86, 95, 96]. Several approaches are currently under evaluation in clinical trials for MSLN‐targeted therapy, including immunotoxins, vaccines, monoclonal antibodies, antibody‐drug conjugates, and adoptive cellular therapies [85].

Chemical signals orchestrating the postsurgical immune system include the increased TNF‐ α and IL‐6 secretion from macrophages [97, 98, 99]. IL‐6 can inhibit T cell infiltration into the TME while promoting cancer progression and invasion [100, 101, 102]. Recent studies have revealed that serum IL‐6 levels might also influence Postoperative cancer outcomes [103, 104] Higher levels of IL‐6 and IL‐6R α in malignant peritoneal fluid compared to those seen in benign conditions and the association between higher peritoneal fluid IL‐10 and VEGF‐A concentrations with poor treatment outcomes provide a rationale for exploring these as therapeutic targets [20, 105, 106]. Further research to examine the applicability of currently available treatment options, such as IL‐6 antagonists, with the intention of re‐calibrating the Postoperative chemical signals can prove beneficial and cost‐effective. Data reflecting the impact of the peritoneal cytokine milieu on immune cell infiltration and functionality indicates that strategies to alter these chemical signals can activate immune effectors locally, with potential abscopal effects on systemic disease outcomes [20, 105].

#### 3.1.1 Immunotoxins

**Immunotoxins**, which are mAbs coupled to toxins, have been designed to limit the therapeutic activity of the toxin to antibody‐targeted tissue [107]. While the therapeutic efficacy of mesothelin targeting immunotoxins has been hampered by the development of neutralizing antibodies, the anti‐MSLN mAb Amatuximab reduces invasive behavior in vitro [108, 109]. Furthermore, an anti‐MSLN antibody‐drug conjugate‐Anetumab Ravtansine exhibited clinical efficacy in a randomized clinical trial of 45 patients with solid tumors [110]. One patient had a complete response, and 5 out of 16 patients with mesothelioma had partial responses. Of note, treatment‐responsive tumors had higher levels of surface mesothelin expression. Epithelial cell adhesion molecule (EpCAM) is upregulated in various solid tumors and modulates cancer progression [111]. EpCAM is being investigated as a potential target for both diagnostic and therapeutic purposes [112, 113].

For example, MOC13PE is an immunotoxin comprised of an EpCAM‐directed antibody linked to a pseudomonas exotoxin‐derivative. MOC13PE gets internalized upon EpCAM binding, and once inside the cancer cell, the toxin arrests protein synthesis, causing apoptosis and immunogenic cell death (ICD) [114]. Intraperitoneal administration of MOC13PE to 21 adults with confirmed EpCAM+ colorectal cancer(CRC) PMD after CRS + HIPEC resulted in a robust local cytokine response coupled with modest systemic effects [115].

#### 3.1.2 Bispecific Antibodies

**Bispecific antibodies** that bind to T cells and engage them with tumor surface antigens to enhance cancer recognition are called Bispecific T cell engagers (BiTEs) [116]. They bind multiple markers concurrently to act as bridges between the T cell receptor (CD3) on T cells and tumor‐associated antigens like EpCAM, to foster the formation of an immune synapse. One such example is Catumaxomab, a tri‐functional bispecific antibody simultaneously binding EpCAM overexpressed on epithelial tumors and CD3 on T cells, as well as the Fcγ receptor on immune effector cells via the antibodies’ Fc domain [117, 118]. Catumaxomab has emerged as a promising therapeutic strategy for malignant ascites and PMD [118, 119]. A clinical trial of 258 patients with malignant ascites demonstrated superior puncture‐free survival outcomes, a composite metric of paracentesis free period and overall survival, with the addition of intraperitoneal catumaxomab to paracentesis [120]. However, a subsequent phase II trial in gastric PMD failed to demonstrate added benefit from catumaxomab when combined with chemotherapy [118]. The efficacy of BiTE therapies in solid tumors has been overshadowed by their limited half‐life and instability in vivo [121]. To overcome these limitations, special delivery platforms such as bisphosphonate‐based‐ calcium alendronate nano‐particle delivery systems have been used to complement a CD3‐HER2 BiTE for the peritoneal ovarian cancer mice models [122].

#### 3.1.3 Antibodies Targeting Growth

Cancer cells preferentially attach to locations near blood vessels or induce angiogenesis with cytokines such as Vascular Endothelial Growth Factor (VEGF) to provide a richer microenvironment for themselves [123]. VEGF has also been implicated in the pathogenesis of ascites, which, in turn, fosters cancer progression and chemotherapy resistance, as part of a self‐reinforcing cycle. As cancer progression relies on angiogenesis, there have been extensive efforts to target VEGF which also plays a critical role in establishing angiogenesis and immunosuppression, both physiologically and in the context of cancer [103, 124]. Tissue analysis of ovarian cancer PMD revealed that higher local concentrations of VEGF were associated with lower levels of CD8 + T cell infiltration in the TME [125]. Although the promising antitumor effects of bevacizumab, an anti‐VEGF mAb, have earned FDA approval for the treatment of PMD, its use may be limited by an increased rate of postoperative wound complications, owing to the critical role VEGF plays in tissue repair and regeneration [126]. A study of 182 colorectal cancer patients undergoing HIPEC revealed significantly higher postoperative morbidity in patients receiving neoadjuvant bevacizumab [127]. A meta‐analysis of 7 randomized clinical trials totaling 5,147 cancer patients calculated the odds ratio of Postoperative wound complications to be 2.32 for patients who received preoperative bevacizumab treatment compared to those who did not [128]. Efforts to reduce the morbidity associated with systemic VEGF blockade have led to the combination of Ramucirumab, an anti‐VEGF receptor‐2 mAb, with a novel local treatment delivery method: pressurized intraperitoneal aerosol chemotherapy (PIPAC) [129]. PIPAC operates on the principle of enhanced local delivery and deeper tumor penetrance of aerosolized therapeutic molecules under pressure [130]. A single‐center retrospective cohort of 50 patients receiving PIPAC for gastric cancer PMD found no association between postoperative complications and ramucirumab [129]. Pathological disruptions in the mesothelial lining following surgery can enhance cancer cell survival by providing a channel into the nutritive environment of the submesothelium [131, 132, 133]. Surgical manipulations should, therefore, consider the risk of mesothelial injury and the subsequent protumorigenic effects [134]. Incorporating immunotherapy strategies that target the attachment and survival pathways in the susceptible peri‐operative period may be a helpful strategy to counteract the enhanced binding opportunities offered by the Postoperative physiology.

#### 3.1.4 Antibodies Targeting the Stroma

To address the challenges posed by the cellular heterogeneity of the TME, increasing efforts are being made to target the stromal components of solid tumors. A recent study from Spain analyzed ovarian and colorectal cancer PMD samples to identify actionable stromal targets for the development of ADCs [135]. Single‐cell RNA sequencing analysis of the mesothelial‐to‐mesenchymal transition that fosters PMD revealed Fibroblast Activation Protein (FAP), Mannose Receptor C type 2, and IL11RA to be potential targets. OMTX705, an anti‐FAP drug conjugate, exhibited superior antitumor activity against in vivo ovarian PMD models compared with placebo and anti‐FAP mAb alone [135, 136].

Recently, members of the B7 class of immunomodulatory molecules have garnered increased interest due to their ability to modify T cell activity within the TME [137, 138, 139]. What sets B7‐H3 apart from other members of the class is its close association with cancer and its supportive stromal components [140]. A recent study revealed B7‐H3 expression to be higher on cancer‐associated stroma compared to tumor cells [140]. A clinical study of 52 patients with peritoneal sarcomas illustrated the safety of intraperitoneally administered I_131_‐omburtamab, a radio‐iodinated anti‐B7‐H3 antibody, when combined with the standard of care [141]. Patients demonstrated superior progression‐free survival and overall survival compared with a historical cohort receiving only the standard of care, prompting the initiation of an ongoing phase II clinical trial [141].

While mAbs certainly have impressive clinical applications, they are unlikely to serve as single‐agent therapies due to their limited efficacy in the setting of antigenically heterogenous solid tumors, capable of antigen editing to allow immune escape. However, they can be employed as complementary approaches to increase the specificity of other treatment modalities, as illustrated by the anti‐folate receptor alpha antibody coupled delivery of photosensitizing substances into micrometastatic peritoneal ovarian cancer cells [142].

### 3.2 Cancer Vaccines

Cancer vaccines employ exogenously modified tumor components to elicit a tumor‐oriented immune response intended to persist over time [143]. The goal is to consolidate this response into immune memory to overcome present, as well as future, tumor challenges [143]. The substrate of cancer vaccines can be derived from autologous or allogeneic tumor cells to be presented as proteins, peptides, or nucleic acids [5]. They can be designed to introduce both patient‐specific and tumor‐specific antigens while utilizing viral, cellular, or chemical carriers to deliver the immunogenic payload [5]. Increasing efforts are being made to target cancer neoantigens, novel epitopes on mutated self‐antigens, due to the specificity they promise [144]. Several clinical trials are underway to test the efficacy of cancer vaccines in the treatment of peritoneal cancers as well as other solid tumors [6].

#### 3.2.1 Tumor Antigen Vaccines

Maveropepimut‐S (formerly DPX‐Survivac) is a lipid‐based vaccine undergoing clinical trials (NCT03836352) for ovarian, tubal, and peritoneal cancer [145]. It relies on generating an educated CD8 + T cell response against the antiapoptotic protein survivin, which is present on gastric, colorectal, and ovarian cancers with minimal normal tissue expression [146, 147]. Despite recruiting heavily pretreated patients with ovarian cancer, phase II trials reported partial responses among 4 of the 19 evaluable patients and a tumor size reduction in 10 of these patients [145]. Several vaccines targeting the Wilms Tumor‐1(WT1) protein are currently being explored in clinical trials (NCT03761914, NCT04739527, NCT04040231, NCT05964361, NCT02649829) for malignancies including malignant pleural mesothelioma, colorectal and ovarian cancer [148, 149, 150].

#### 3.2.2 Personalized Vaccines

Vigil (Gemogenovatucel‐T) is a novel cancer vaccine generated by irradiating and modifying autologous surgical specimens to elicit a tumor‐specific host immune response [151]. While the antigens from the irradiated tumor cells serve as the trigger for the immune recognition of residual cancer, immunomodulatory genetic material introduced into these cells, namely the RNA encoding GM‐CSF and shRNA downregulating the immunosuppressive chemokine TGF‐β, make the TME more available for immune effector cell infiltration [152]. A meta‐analysis of 7 randomized clinical trials involving a total of 322 patients with advanced ovarian, tubal, and peritoneal cancer has revealed that Vigil exhibits safety and superior survival outcomes compared with placebo [151]. A clinical trial (NCT02346747) is currently exploring the efficacy of Vigil as a complement to the standard treatment protocols for gynecologic malignancies.

#### 3.2.3 mRNA Vaccines

The efficacy of COVID‐19 mRNA vaccines has reinvigorated the study of lipid nanoparticles(LNP) as a stable platform for the delivery of mRNA vaccine immunotherapies [153]. mRNA are noninfectious particles with a low risk of mutability, and they can direct the host cell towards the production of the desired proteins and modulate the immune response [154]. For example, an mRNA vaccine promotes neo‐antigen specific T cell infiltration into the tumor and subsequently prolong survival in pancreatic adenocarcinoma(PDAC) patients [155]. Individualized neo‐antigen mRNA have been cloned, as identified by comparative tumor and healthy tissue genome sequencing and administered to patients after surgery as part of a phase I clinical trial with promising results [155]. Patients who were able to generate a detectable T‐cell response to the targeted peptides after the vaccine had superior survival [155]. This trial demonstrates the exciting possibility of designing individualized cancer vaccines within weeks that can be administered safely following surgery [155].

#### 3.2.4 Dendritic Cell Vaccines

Several clinical trials are introducing mRNA vaccines to autologous dendritic cells ex vivo, allowing them to process and present the encoded tumor antigens to T cells after infusion [153, 156]. A clinical trial from the Netherlands for malignant peritoneal mesothelioma utilized *ex vivo* expanded dendritic cells that had been cultured in the presence of an allogeneic tumor to direct the immune effector cells against residual tumor [157]. The treatment, MesoPher, administered solely to patients who had received CRS + HIPEC for malignant peritoneal mesothelioma, proved safe, and among 16 evaluable patients, the median PFS was 12 months [157]. Notably, a study from another Dutch cohort reported median PFS post‐CRS‐HIPEC, including incomplete CRS, to be 23.1 months [158]. This indicates that MesoPher could provide an additional 11 months of PFS to these patients.

Clinical trials (NCT05920798) are also underway to evaluate the safety and efficacy of a folate receptor alpha‐loaded dendritic cell vaccine, FRaDC, for the treatment of recurrent ovarian and peritoneal cancer [159].

**Figure 2:**
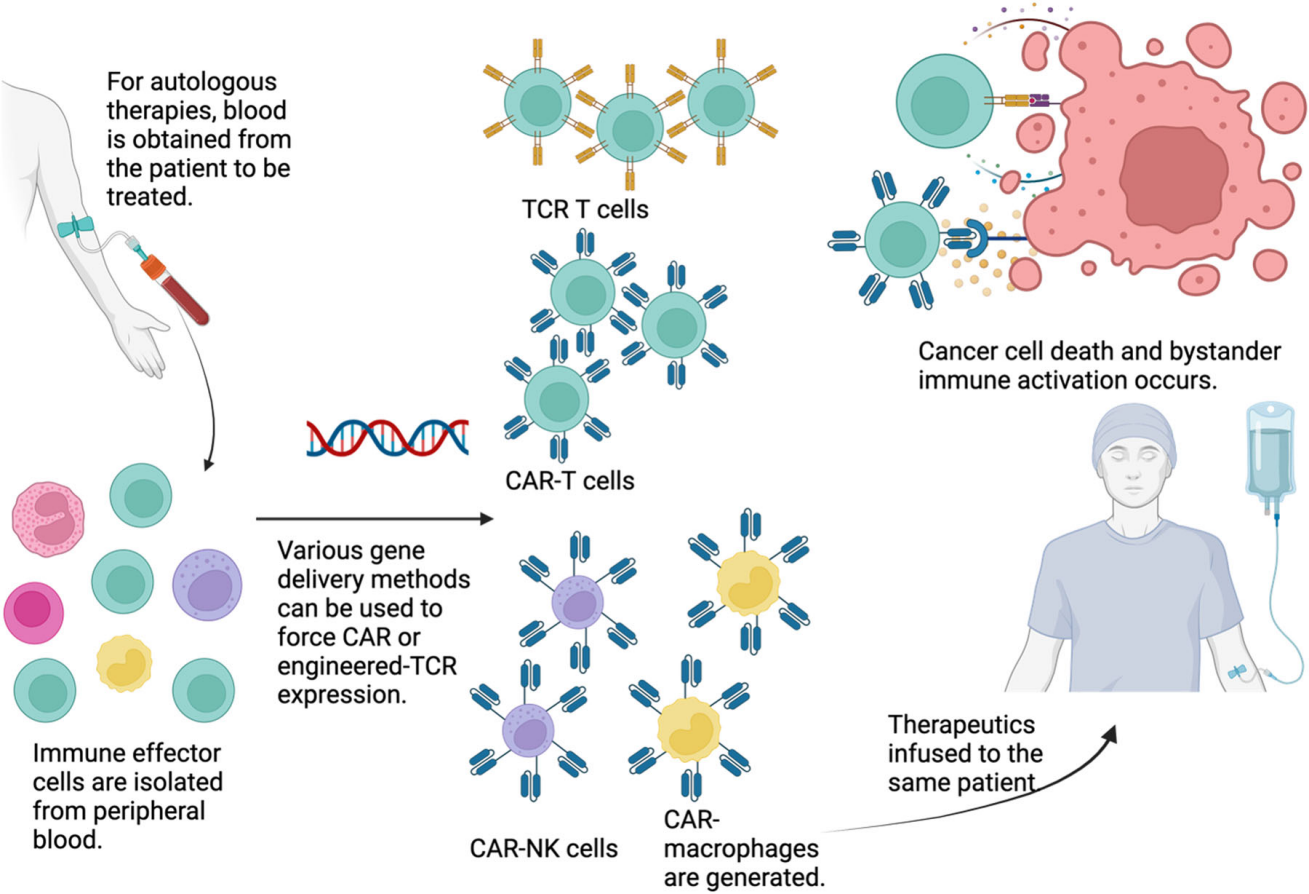
Adoptive Cell Therapies for Peritoneal Cancer.

The major discrepancy between the in vitro success in generating tumor‐specific effector responses with cancer vaccines and the suboptimal clinical efficacy can partly be explained by the limited immune effector infiltration into the TME [160, 161]. Interventions to enhance vaccine‐educated effector cell recruitment and activity in the TME are needed to ensure the successful clinical translation of in vitro results [162]. To that end, administering immunotherapies during times of augmented immune recruitment to the desired site should be considered. The peri‐operative period can provide a unique window of opportunity to establish synergistic cooperation between immunotherapies and the inherent immune system (Figure 2).

### 3.3 Oncolytic Virus Therapy

Oncolytic viruses (OV) preferentially infect and/or replicate in cancer cells through selective tropism that can be natural or engineered [163]. Several processes governing cancer biology, such as the compromised immune response or the exaggerated antiapoptotic signals, can be manipulated to ensure the specificity of OV [163]. However, systemically administered OV must avoid neutralization by pre‐existing or therapy‐induced antiviral antibodies to reach the formidable TME [164, 165]. Hence, most OV are injected intratumorally, and the subsequent abscopal effects help clear distant disease [166]. Applying OV locally to the peritoneum during surgery could be a helpful strategy.

Eradication of virally infected cells relies on Type I IFN secretion that upregulates viral antigen expression on the cell surface major histocompatibility complex (MHC) for the subsequent immune activation [167, 168, 169, 170]. To continue replicating, viruses must tailor mechanisms to counteract this response, which could otherwise result in the elimination of the infected cell [170]. Viruses with natural or engineered sensitivity to IFN are preferentially employed as OV to enhance viral replication in cancer cells with defective IFN secretion while immune competent cells remain uninfected [171, 172]. Several OVs have also been designed to rely upon cellular machinery characteristically hyperfunctional in cancer cells, such as the antiapoptotic pathways [165].

Oncolysis, OV‐induced tumor cell lysis, is a form of immunogenic cell death that attracts immune effectors through damage‐associated molecular patterns (DAMPs) and the release of viral products [173, 174, 175]. Tumor lysis can then trigger the exposure of tumor‐associated antigens that educate a tumor‐specific immune response [173, 174, 175]. The released viral products can either be viral progeny, which propagates the infectious cycle and oncolysis in other cancer cells, or noninfectious particles, capable of immune recruitment [173, 174, 175]. Additionally, virus‐induced dendritic cell maturation and the release of T cell supportive cytokines promote an immune reaction toward the surrounding uninfected cells [176]. Finally, the antiviral and antitumor immunity, primed and educated by the local reaction, can join the systemic circulation [173, 177]. As a result, even if the OV had been administered intratumorally, the immune response can reach distant sites to propagate the antitumor effects systemically [173, 177].

The benefits of oncolytic virotherapy can extend beyond the preferential infection of diseased cells. Even when stripped of their replication machinery that enables oncolysis, as is the case for helper‐dependent viruses, OVs can still act as Trojan horses for the selective delivery of a large cargo of cancer modulatory genes [178]. These therapeutic genes can come in the form of cytokines, chemokines, costimulatory molecules, immune checkpoint inhibitors, or immune effector engager molecules such as BiTEs and can potentiate adoptive cellular therapies as well as endogenous host immune cells [179]. Of the 4 oncolytic viruses approved for marketing worldwide, only talimogene laherparepvec (T‐VEC), an attenuated oncolytic HSV‐1 encoding GM‐CSF, holds FDA approval [165].

in a recent phase I clinical trial, the injection of an oncolytic adenoviral platform expressing IL‐12 and a PD‐L1 blocking antibody, CAdVEC, into the primary tumor resulted in regression of metastases [180].

Intraperitoneal injection of an oncolytic human recombinant adenovirus, H101, that selectively replicates in cells with p53 dysfunction, has demonstrated safety and efficacy in local immune activation [181]. Of note, in a study of 40 patients, 5 had complete resolution of ascites, and treatment was associated with increased immune infiltrate in the peritoneal fluid. Taken together, these results suggest that the peritoneal tumor microenvironment (TME) is amenable to manipulation with oncolytic virotherapy.

To further exploit the enhanced immune recruitment, oncolytic viruses can be utilized to lead to the expression of molecules that can tilt the balance towards an antitumor effect. One such example is an oncolytic adenovirus EnAdenotucirev (EnAd), engineered to cause the secretion of CD3‐EpCAM BiTE molecules from the infected cancer cells (EnAd BiTE) [182, 183]. EnAd BiTE was able to elicit a robust immune response in vitro when malignant ascites patient samples containing tumor cells were cocultured with healthy donor T cells.

CF33‐hNIS‐antiPDL1 is an oncolytic vaccinia virus expressing human sodium iodide symporter and anti‐PDL1 antibody. Patient‐derived xenograft mice with gastric PMD showed prolonged survival, reduced tumor burden, and decreased incidence of ascites when treated with CF33 compared with placebo [183]. CF33 and its derivatives have been approved for phase I clinical trials (NCT06063317, NCT05346484, NCT05081492) for the treatment of solid tumors.

Host immunity against the viral vector frequently presents a significant challenge for oncolytic virotherapy as many of the viral vehicles will be recognized by the patient's immune system [184]. Nonetheless, the 94% disease control rate achieved by the intratumoral administration of an immunomodulatory oncolytic adenovirus, LOAd703, to patients with metastatic pancreatic cancer underscores the potential local delivery options hold, despite antiviral immunity [185]. LOAd703 is now being used in clinical trials (NCT03225989) against colorectal and ovarian carcinoma as well.

Intraperitoneal administration of OV can similarly bypass the neutralizing antibody and complement responses available systemically [186]. Intraperitoneal administration may also allow enhanced access to the portal circulation, compared with the more conventional intratumoral route [187, 188].

Despite covering a surface area almost as wide as the skin (1.8 m^2^, peritoneum to 2 m^2^, skin), the peritoneum normally receives only 1–2% of the cardiac output [188, 189]. While this discordance might contribute to low peritoneal delivery of systemic treatments, stimuli such as local hyperthermia introduced in the form of Hyperthermic Intraperitoneal Chemotherapy (HIPEC) can cause vasodilation and enhance blood flow [190]. Additionally, studies that have revealed an increase in the uptake, replication, and transgene expression of oncolytic viruses during hyperthermia might provide a rationale for combining HIPEC with oncolytic virotherapy [191].

### 3.4 Adoptive Cell Therapy

Adoptive Cell Therapy is the administration of autologous or allogeneic cells that have been manipulated *ex vivo* [7, 192, 193]. Adoptive cellular therapies can be designed to replace, modify, or even surpass the functions of the host's endogenous immune system, particularly during states of compromise or dysfunction, due to cancer or surgery. in addition to replenishing the endogenous host immune system, current treatment modalities can reprogram the dysfunctional cells or redirect their focus toward a desired target.

The immune response to surgery is characterized by an initial pro‐inflammatory surge followed by a longer suppressed state [194, 195, 196, 197]. Immediately after surgery, circulating neutrophils, monocytes, and lymphocytes increase as peripheral Natural Killer (NK) cells decrease [42, 198]. Additionally, these cells experience substantial shifts in functionality as evidenced by a change in their phenotype, which can be quantified by alterations in cell surface markers [197, 199].

The acute inflammatory response to surgery increases the circulating neutrophils within 24 h [197, 200, 201, 202]. Neutrophils prevent bacterial colonization of the surgical site and initiate the inflammatory cascade intended to promote wound healing [203]. Cancer impairs neutrophil maturation, potentially favoring a pro‐tumor inflammatory response in the post‐cancer surgery setting [204, 205]. Neutrophils also create protein‐coated webs of nucleic acids, Neutrophil Extracellular Traps (NETs), in response to DAMPs from surgical trauma [206, 207]. NETs promote tumor progression by binding to circulating tumor cells(CTCs), protect CTCs from immune recognition, aid extravasation and metastasis [208, 209, 210]. Moreover, recent animal studies suggest that the migration of neutrophils out of inflamed tissue can induce tumor progression and the proliferation of pre‐neoplastic cells in the tissue surrounding the surgical trauma [211]. Recombinant cytokine and chemokine treatments that are already in use for other indications might be employed in the setting of cancer surgery to control such protumorigenic effects of neutrophil chemotaxis should the same effects on cancer cells be demonstrable in “humanized” systems.

A notable study on colorectal cancer patients revealed that NK cells, responsible for innate antitumor immunity, are functionally and quantitatively compromised up to 8 weeks post‐surgery [195, 212]. in an immunocompetent host, NK cells recognize and kill dysfunctional cells, including cancer cells, that can go undetected by immune surveillance mechanisms that rely upon MHC‐restricted recognition [213]. Recognizing the association between compromised NK cell function with an increased propensity for the development of cancer in humans, some groups have utilized cytokine‐derived therapies to restore NK cell function in peritoneal cancers [214]. The gap between the significant antitumor effects of NK cells and compromised functionality in cancer patients has suggested that modified NK cells could serve as augmented therapeutic platforms. Oncolytic viruses have also been tailored to stimulate NK cells and myeloid cells by releasing stimulatory cytokines, such as IL‐12, in the context of peritoneal carcinomatosis [215, 216, 217, 218, 219]. Treatment modalities that engage multiple components of the immune system to tilt the overall balance toward an antitumor response can prove particularly useful in the post‐surgery setting where several effectors work to promote tumor progression.

Surgical stressors decrease the total lymphocyte count within 24 h [196, 197]. Postoperative T cells have compromised functionality, marked by diminished Interferon‐ γ (IFN‐ γ) secretion [43, 220]. Most circulating T cells are PD‐1 + TIM‐3 + , which are regarded as “exhausted” T cells. While debates are ongoing for such a definition, efforts are being made to rescue T cells from this state [221, 222, 223]. Although almost all subpopulations of T cells are suppressed within the immediate postoperative period, there are conflicting data on trends regarding the T‐regulatory cells (Tregs), the immunosuppressive fraction of T cells [224]. On postoperative day 6, peripheral Tregs constitute a higher proportion of the T cell population than before, suggesting that normal total T cell counts are not accurate indicators of cytotoxic antitumor function [225].

The most widely studied examples of augmented cellular therapies include Tumor Infiltrating Lymphocytes (TILs), T cell receptor‐modified T cells (TCRs), and Chimeric Antigen Receptor (CAR) modified cells. The disappointing discrepancy between the robust in vitro antitumor effects of tumor‐infiltrating lymphocytes (TILs) and the poor in vivo activity underscores the immunosuppressive impact of the TME [226, 227, 228]. Armed with a deeper understanding of the inhibitory mechanisms governing the TME, researchers are now exploring a variety of superior cellular therapies that aim to overcome these obstacles.

CARs are an impressive tool for coupling immune effector function with specific target engagement [229]. CARs consist of an engineered extracellular moiety that recognizes the desired ligand, coupled with intracellular signaling and costimulatory domains that activate the CAR‐expressing cell upon target‐antigen binding [230]. While NK cells, NKT cells, and perhaps macrophages can also be modified to express a CAR, until recently the focus has been on CAR‐T cells [231, 232, 233]. Having achieved unprecedented results with hematologic malignancies, a plethora of CAR‐T cells are now being rigorously tested against solid tumors. Despite presenting a potent and highly modifiable platform that can be tailored to target any tumor‐associated antigen (TAA), CAR‐T cells have had limited success in treating solid tumors [234, 235]. The heterogeneous and dynamic expression of target antigens and the immunosuppressive TME behind the physical stromal barrier have constrained their clinical efficacy in comparison to hematological malignancies [236]. The associated toxicities that can lead to reversible organ dysfunction, notably Cytokine Release Syndrome (CRS) and Immune‐Effector Cell Associated Neurotoxicity Syndrome (ICANS), have also raised concerns about their clinical applications [237]. The focus of the field has shifted toward overcoming these challenges.

To overcome the heterogenous TAA expression, efforts have been made to identify tumor antigen‐agnostic targets for cancer immunotherapy. Aberrant glycosylation patterns have emerged as promising targets while serving as markers for cancer and cancer‐associated fibroblasts that can be expressed on multiple molecules on the cell surface [238, 239, 240]. Certain overexpressed variants of MUC‐16 have glycosylation patterns that can be bound and neutralized for therapeutic purposes [241, 242, 243]. Since downregulation of MUC16 in PDAC xenograft models results in hampered tumor progression and invasion, targeting these aberrant glycans to prevent PMD can be successful [244]. Guiding an immune effector towards these patterns by coupling a glycan‐binding molecule, such as a lectin, to cytotoxic function can be a viable approach [245, 246]. Indeed, our work has demonstrated that CAR‐T cells expressing a banana‐derived lectin can target solid tumors known to progress to PMD [247]. Another glycan‐targeting therapeutic is a CAR‐T cell designed to recognize the Tumor‐Associated Glycoprotein‐72 (TAG72), a carbohydrate antigen commonly found on multiple cell surface molecules of epithelial adenocarcinomas [248]. Clinical trials have demonstrated the safety of TAG‐72 CAR‐T cells for CRC patients and mouse models of peritoneal ovarian cancer suggest that their intraperitoneal administration might confer a survival benefit [249].

One approach to counteract the suppressive signals of the TME has been to armor CAR‐T cells with additional immunostimulatory capabilities. MUC16 CAR‐T cells engineered to secrete IL‐12 demonstrated superior efficacy in mouse models of ovarian PMD compared with conventional MUC16 CAR‐T cells and are currently being tested in a phase I clinical trial (NCT02498912) delivered by IP injection [250, 251]. in addition, the efficacy of various anti‐mesothelin CAR‐T cell therapies for the treatment of multiple solid tumors, including peritoneal malignancies, is being explored in clinical trials worldwide [252, 253].

Most current adoptive cell therapies are armored with ICB‐like molecules to ensure maximum therapeutic efficacy in the face of immunosuppressive barriers erected by the TME. HER2 CAR‐T cells and HER2 CAR macrophages are being used in clinical trials to treat solid tumors, including PMD (NCT04684459, NCT03740256, NCT04511871, NCT04660929) [254, 255]. To better prepare them for the challenges within the peritoneal TME, an immunomodulatory switch receptor has been incorporated into HER2 CAR‐T cells [256]. This receptor can “switch” the normally suppressive external PD‐L1 signal into an internally proliferative 4‐1BB signal, hence turning the immunosuppressive signals in the malignant ascites TME into stimulatory ones for the therapeutic agent [256]. Incorporating the PD‐L1 switch receptor significantly improved the therapeutic efficacy of locally delivered HER2 CAR‐T cells in mice; this protocol is now being tested in clinical trials for PMD (NCT04684459).

Surviving systemic circulation, homing to the tumor site, and infiltrating the physical and immune barriers of the TME is no easy feat. When faced with the challenges of eradicating solid tumors, adoptive cell therapies struggle to replicate the remarkable efficacy with which they eliminate hematological malignancies. Surgically guided strategies can augment immunotherapeutic efficacy by mitigating interference within the systemic circulation and moving past the barriers of the inhospitable TME. in conjunction with systemic treatment approaches, targeted delivery of cell‐based therapies can also bypass the premature cellular “exhaustion” induced by improper overstimulation, allowing the cells to reach their intended site of action with optimal functionality [64]. Designing protocols to bypass the systemic circulation and deliver adoptive cell therapies locally into areas surgically stripped of desmoplastic stroma can potentially enhance the efficacy of CAR‐T cells and other adoptive cellular therapies. Preclinical studies in mice with anti‐carcinoembryonic antigen (CEA) CAR‐T cells demonstrated that IP injection results in a more rapid response to therapy and prolonged protection against tumor rechallenge, while also improving the immune cell composition in the TME [65, 257]. Preliminary data from subsequent clinical trials confirmed that CEA CAR‐T cells administered via the intraperitoneal route provide superior disease control [258]. This superior local and systemic activity, along with long‐term efficacy, has also been observed in other solid tumors with regionally administered CAR‐T cell therapies compared to systemic administration [64]. Furthermore, an in vivo study of PMD demonstrated that delivering IL‐12 mRNA ‐engineered tumor specific CD8 + T cells via the intraperitoneal route can overcome the limitations of systemic IL‐12 administration and produce a more favorable immune profile in the TME [259].

Although most therapeutic efforts have employed T cells, other effector cells can also be adoptively transferred. To mitigate the associated cytokine release syndrome and overcome the physical barriers that block tumor infiltration by CAR‐T cells, CAR‐NK cells, and CAR‐macrophages have been employed in in vivo models of PMD [260]. Peritoneal macrophages derived from patients with malignant ascites modified to express a HER2 CAR have provided superior control of PMD associated with gastric cancer in mice and enhanced infiltration of elements of the adaptive immune system into the TME.

While it might be possible to design “omnipotent cells” with exceptional antitumor activity in silico, the risk and severity of collateral adverse effects increases as cells are engineered further from their natural genomic state. The goal is to minimize laboratory manipulations while optimizing therapeutic effectiveness. Researchers are now using mRNA platforms to eliminate the need for ex vivo modifications. Lipid nanoparticles (LNPs) can be tailored to ensure uptake by specific cells, as demonstrated by a targeted approach delivering mRNA instructions for the expression of a Glypican‐3 targeting CAR to hepatic macrophages [261]. The transient effects of mRNA therapeutics, incapable of genomic integration, mitigate the safety concerns associated with highly modified cell therapy products [262]. Moreover, in situ programming of immune cells with mRNA LNPs eliminates the need for ex vivo manipulation, reducing variability and cost while enhancing efficacy.

Several constraints to the clinical efficacy of CAR‐T cell therapeutics can be overcome by CAR‐NK cells. Unlike T cells, NK cells do not require prior sensitization to engage with a target, enabling them to recognize a broader range of pathologies [263]. An NK cells’ response to any interaction will be determined by the balance between the stimulatory and inhibitor signals from surface receptors. This allows us to fine‐tune and target the activity of engineered NK cells [264]. Moreover, NK cells are not MHC‐restricted, making NK cell‐based therapies safe and efficacious even when allogeneic. This enables the production of ‘off‐the shelf’ NK cells that can respond rapidly to the patients’ needs. Multiple CAR‐NK cells are being explored for malignancies affecting the peritoneum, with several in clinical trials.

Natural Killer group 2 member D (NKG2D), a receptor naturally expressed on multiple immune cells, will favor the activation of the NK cell when interacting with one of its ligands (NKG2DLs) which are upregulated in cancer but rarely expressed on healthy tissue [265]. Autologous and allogeneic CAR‐NK cells that can recognize NKG2DLs have been intraperitoneally administered to chemotherapy‐resistant CRC patients to achieve marked reduction in ascites volume and tumor cell count in ascitic fluid. 1 of the 3 patients also received ultrasound guided percutaneous liver injections that resulted in the regression of hepatic metastases. No serious adverse events were observed (all events grade 0‐2). Phase I/II Clinical Trials (NCT05922930) are testing the intraperitoneal injection of IL‐15 transduced Trophoblast Surface Antigen‐2 (TROP‐2) CAR NK cells against ovarian and pancreatic cancer [266]. c‐Met, GPC3, Mesothelin, PSCA and FR α CAR‐NK cells have also shown success against hepatocellular and pancreatic carcinoma models [267, 268, 269, 270, 271, 272].

### 3.5 Delivery Systems

Efforts to improve the efficacy of therapeutic agents by facilitating their infiltration into the solid tumor microenvironment have guided the design of novel delivery platforms that can be administered systemically and locally [8]. These platforms employ materials or cell‐based carriers to ensure targeted delivery, protecting therapeutics from undesired interactions that may compromise stability and lead to drug‐related adverse events [273].

Hydrogels are biomaterial delivery systems that resemble the extracellular scaffold of the tumor‐supportive stroma [274, 275]. Hydrogels retain high volumes of water and offer locally injectable highly malleable platforms [274, 275]. in addition to their potential immunoadjuvant properties, they can be tailored to harbor multiple immunomodulatory components with enhanced stability [276]. For example, when sulfasalazine was delivered using a hydrogel‐based system, the TME of hepatocellular carcinoma in mice with malignant ascites exhibited an antitumor immune profile [277].

Nanoparticles, 10‐100 nm carriers capable of precise payload delivery through their chemically and physically engineered affinities, can be broadly classified into 2 groups: organic (such as liposomes and micelles) and inorganic (such as gold and silica) [278, 279]. A notable example is the engineered apoptosis bio‐inspired nanoparticle that has been used as a vehicle for the in vitro delivery of the histone deacetylase (HDAC) inhibitor, TMP195, to peritoneal resident macrophages [280]. in this system, molecular surface markers associated with apoptotic bodies have been used as substrates for nanoparticle vehicle design, selectively enhancing macrophage recognition of the drug. While nanoparticles offer a modifiable delivery platform with inherent immunostimulatory effects, concerns remain regarding their abilities to infiltrate solid tumors consistently and efficiently [281].

Cell‐based delivery systems encompass a spectrum, from simply conjugating immunotherapeutics with cellular components to employing genetically modified cells as carriers [282, 283, 284]. These designs serve multiple purposes: They can shield therapeutics from immune recognition by incorporating natural cellular components like membranes and exosomes, enhance stability and solubility, or facilitate infiltration into the TME by camouflaging the payload with tumor‐derived components.

Exogenous cytokines may also be delivered by cell‐based vehicles, either by forced expression in transgenic cells or by using the cell to encapsulate mRNA or the relevant protein. For example, the reduction in IL‐2 levels after surgery has prompted several clinical trials to explore the benefits of its replacement [285, 286, 287, 288]. Patients with renal, pancreatic and colorectal cancer who received preoperative IL‐2 had improved prognostic outcomes and experienced minimal treatment‐related adverse events [289, 290, 291]. These studies highlight the potential long‐term benefits of simple immunomodulatory strategies when employed pre‐operatively [292]. Nonetheless, wider use of recombinant IL‐2 has been limited by its low stability and relatively high risk of toxicity when administered systemically [293, 294, 295]. Clinical studies demonstrating the better tolerability of intraperitoneal IL‐2 by cancer patients could inform the design of peri‐operative immunotherapy protocols [296, 297]. The search for a non‐tumorigenic cell‐based delivery system to enhance drug stability led to the engineering of encapsulated Retinal Pigmented Epithelial cells expressing IL‐2 [298]. in vivo studies have proven this system's scalability and efficacy in ensuring sustained higher local concentrations with lower toxicity when delivered IP to treat ovarian peritoneal carcinomatosis. The superior local T cell activation achieved by the encapsulated cell‐based system resulted in the establishment of immune memory capable of warding off tumor recurrences, as proven by tumor rechallenge experiments in mice.

While research on novel delivery platforms promises to elevate the efficacy of cancer immunotherapy, protocols with optimal specificity and tolerability still require orchestration by surgeons. The war between cancer and peri‐operative immunity can only be resolved by surgeons armed with the skills and knowledge to deliver treatments precisely and timely (Table 1).

**Table 1:** List of Immunotherapeutic Agents Targeting Peritoneal Cancer and Associated Tumors Prone to Peritoneal Metastasis Referenced in the Text.

Group	Type	Therapeutic	Target	Stage	Reference
Monoclonal Antibodies	Monoclonal Antibody(MAb)	Amatuximab	Mesothelin	Completed and Terminated Clinical Trials	[108, 299]
	Antibody Drug Conjugate(ADC)	Anetumab Ravtansine	Mesothelin	Active Clinical Trials	[109, 110, 300, 301]
	Bispecific T Cell Engager (BiTE)	Catumaxomab	EpCAM and CD3	Active Clinical Trials	[118, 120]
	Immunotoxin	MOC31PE	EpCAM	Completed Clinical Trials	[115, 302]
	MAb	Bevacizumab	VEGF	FDA Approved for solid tumors	[126, 127, 128, 303]
	MAb	Ramucirumab	VEGF	FDA Approved for solid tumors	[129]
	ADC	OMTX705	FAP	Preclinical studies	[135, 136]
	Radiolabeled MAb	I^131^‐omburtamab	B7H3	Active Clinical Trials	[141]
Vaccines	Cancer Vaccine	Maveropepimut‐S	Survivin	Active Clinical Trials	[145]
	Cancer Vaccine	Gemogenovatucel‐T	Autologous tumor	Active Clinical Trials	[151, 152]
	Dendritic Cell Vaccine	MesoPher	Allogeneic mesothelioma cells	Active Clinical Trials	[157]
	Cancer Vaccine	WT‐1 vaccines (peptide and dendritic cell vaccines)	WT1	Active Clinical Trials	[148, 149, 150]
	Dendritic Cell Vaccine	FRaDC	Folate Receptor‐ α	Active Clinical Trials	[159]
Oncolytic Viruses	Adenovirus	H101	p53 dysfunction	Active Clinical Trials	[181]
	Adenovirus	EnAdenotucirev BiTE	EpCAM and CD3	Preclinical studies	[182]
	Vaccinia Virus	CF33‐hNIS‐antiPDL1	PDL1	Active Clinical Trials	[183]
	Adenovirus	LOAd703	—	Active Clinical Trials	[185]
Adoptive Cell Therapy	CAR‐T cell	TAG‐72 CAR‐T cells	TAG‐72	Active Clinical Trials	[248, 249]
	CAR‐T cell	IL‐12 secreting MUC16 CAR‐T cells	MUC16	Active Clinical Trials	[250, 251]
	CAR‐T cell	Anti‐Mesothelin CAR‐T Cells	Mesothelin	Active Clinical Trials	[252, 253]
	CAR‐T cell	HER2 CAR‐T Cells	HER2	Active Clinical Trials	[254, 256]
	CAR‐Macrophage	HER2 CAR Macrophages	HER2	Active Clinical Trials	[255, 260]
	CAR‐T cell	CEA CAR‐T Cells	CEA	Active Clinical Trials	[257, 258]
	Tumor Specific T cells	IL‐12 mRNA‐engineered CD8 + T Cells	Tumor‐specific	Preclinical studies	[259]
	CAR‐Macrophage	Glypican‐3 CAR Macrophage	Glypican‐3	Preclinical studies	[261]
	CAR‐NK	NKG2D CAR‐NK cells	NKG2DL	Active Clinical Trials	[265]
	CAR‐NK	IL15 transduced TROP2 CAR‐NK cells	TROP2	Active Clinical Trials	[266]
	CAR‐NK	c‐Met CAR‐NK	c‐MET	Preclinical studies	[267]
	CAR‐NK	Glypican‐3 CAR NK	Glypican‐3	Active Clinical Trials	[268]
	CAR‐NK	Mesothelin CAR‐N K	Mesothelin	Active Clinical Trials	[270]
	CAR‐NK	Prostate Stem Cell Antigen (PSCA) CAR‐NK	PSCA	Preclinical studies	[271]
	CAR‐NK	Folate Receptor α (FR α) CAR‐NK cell	FR α	Preclinical studies	[272]
Delivery Systems	Hydrogels	Sulfasalazine delivered via hydrogel	TME modification	Preclinical studies	[277]
	Nanoparticles	HDAC inhibitor TMP195 delivered via Apoptosis bio‐inspired nanoparticles	Immunomodulation	Preclinical studies	[280]
	Cell based systems	Retinal Pigmented Epithelial cells expressing IL‐2	Immunomodulation	Preclinical studies	[298]

## 4 Concluding Remarks

Effective surgical treatment of peritoneal tumors can begin before the patient enters the operating room. Harnessing the power immunotherapeutics have to prime the immune system pre‐operatively can help maximize the efficacy of the multidisciplinary treatment of cancer. Manipulating the immune system for therapeutic success during the unique postoperative period, characterized by the lowest tumor burden and enhanced tumor antigen availability, can offer a valuable opportunity. A competent immune system exposed to the increased antigen exposure of surgery can produce comprehensive immunologic memory that serves as a gatekeeper to prevent disease recurrence. However, any perioperative immunomodulatory strategy should weigh the risk of potential tumor progression against increased susceptibility to infection by immune suppression, wound and anastomosis complications due to disordered growth, vascular events, and organ compromise resulting from inflammation.

For cancers of the peritoneum, intraperitoneal delivery of immunotherapies, possibly in combination with HIPEC, can enhance treatment efficacy and circumvent some of the barriers set by the immunosuppressive TME. Furthermore, the activated perioperative immune response in patients with compromised immunity at baseline, coupled with the enhanced antigen exposure and low tumor burden following surgery presents a unique window of opportunity. Efforts to better characterize this period can pave the way for focused protocols aimed at leveraging its potential.

## Synopsis

This review explores the mechanisms driving cancer progression and treatment resistance in the peritoneal cavity to identify potential therapeutic targets. The aim is to examine the possible synergistic benefits of combining immunotherapy with surgical approaches.

## Data Availability

Data sharing not applicable to this article as no datasets were generated or analysed during the current study.
